# PIPTO: Precise Inertial-Based Pipeline for Threshold-Based Fall Detection Using Three-Axis Accelerometers

**DOI:** 10.3390/s23187951

**Published:** 2023-09-18

**Authors:** Stavros N. Moutsis, Konstantinos A. Tsintotas, Antonios Gasteratos

**Affiliations:** Department of Production and Management Engineering, Democritus University of Thrace, 12 Vas. Sophias, GR-671 32 Xanthi, Greece; ktsintot@pme.duth.gr (K.A.T.); agaster@pme.duth.gr (A.G.)

**Keywords:** human fall detection, acceleration-based recognition, wearable device

## Abstract

After traffic-related incidents, falls are the second cause of human death, presenting the highest percentage among the elderly. Aiming to address this problem, the research community has developed methods built upon different sensors, such as wearable, ambiance, or hybrid, and various techniques, such as those that are machine learning- and heuristic based. Concerning the models used in the former case, they classify the input data between fall and no fall, and specific data dimensions are required. Yet, when algorithms that adopt heuristic techniques, mainly using thresholds, are combined with the previous models, they reduce the computational cost. To this end, this article presents a pipeline for detecting falls through a threshold-based technique over the data provided by a three-axis accelerometer. This way, we propose a low-complexity system that can be adopted from any acceleration sensor that receives information at different frequencies. Moreover, the input lengths can differ, while we achieve to detect multiple falls in a time series of sum vector magnitudes, providing the specific time range of the fall. As evaluated on several datasets, our pipeline reaches high performance results at 90.40% and 91.56% sensitivity on MMsys and KFall, respectively, while the generated specificity is 93.96% and 85.90%. Lastly, aiming to facilitate the research community, our framework, entitled PIPTO (drawing inspiration from the Greek verb “π$\overset{´}{\iota }$πτω”, signifying “to fall”), is open sourced in Python and C.

## 1. Introduction

Following traffic collisions, the World Health Organization reports that human falls are the second most common cause of accidental deaths, resulting in approximately 684,000 annual fatalities [1]. Each year, over 37.3 million deadly falls are recorded, with the highest rates corresponding to individuals over 60 who live in nations with low- and middle-income backgrounds. As the elderly are prone to falling easily and more frequently, and their recovery process is usually more challenging and time consuming, the research community dedicates its attention to detecting human falls [2,3,4] in places where older people live, e.g., residential health facilities [5] and nursing homes [6,7]. Additionally, a fall detector is applied in workplaces in order to enhance employees’ safety [8]. Therefore, this task is one of the most rising research challenges, owing to the fact that accident determination can be life saving if combined with a call-for-help or an alarm system [9].

Aiming to address this issue, the existing approaches vary in terms of used sensors, such as ambiance, wearable, or a combination of them [10,11,12,13], as well as on the techniques which are applied [14,15], such as machine learning [16,17,18] and heuristic [19]. In particular, wearable devices, e.g., accelerometers, gyroscopes, barometric sensors, magnetometers and electromyographic sensors [20,21,22], are those which fit on an individual and can generate data directly from the observed subject. On the other hand, ambiance sensors, e.g., red-green-blue (RGB) mono or stereo cameras, and microphones receive stimuli from the environment, and accidents are detected by processing the data from the subject’s external surroundings. Although the latter can offer high performance while, at the same time, detecting accidents occurring in more than one individual, they also present notable drawbacks [23]. More specifically, as data are received from particular territories, an occurring event may not be detected in an area not included in the sensor’s range. To reduce and deal with these restrictions, some frameworks employ the sensors on patrol robots [24,25]. Yet, the risk of either not detecting an event or recognizing it with delay persists. This limitation is of particular significance, as prompt detection is of the utmost importance in cases of older people, while it is only helpful if seen by the system immediately [9,26]. On the contrary, when a fall detector operates with high-frequency data provided through a wearable device, these limitations do not exist, as the sensor continuously receives information from the individual [27]. Additionally, the acceleration input directly provides human movements and activities [28]. A typical example includes the case of the LPMS-B2 sensor, which transmits data up to 400 Hz (https://www.lp-research.com/9-axis-bluetooth-imu-lpmsb2-series/ (accessed on 10 September 2023)), as it was set at 100 Hz on the creation of KFall [21]. To this end, it is evident that an event is almost impossible to miss if this type of sensor is employed.

Regarding the techniques applied for recognizing a human fall, machine and deep learning approaches are the ones that attracted the research community, mainly due to their improved accuracy. However, some negative aspects still exist [27], mainly concerning their model training process and the computing power needed. Moreover, most of these models, i.e., support vector machines (SVMs), *k*-nearest neighbor (*k*-NN), decision trees, and random forests, which are usually used, do not accept dynamical inputs [29]. As a result, each input should have the same length. In the case of the deep learning models, i.e., multilayer perceptrons (MLPs) [30] and convolutional neural networks (CNNs) [7], the majority also require consistent input dimensions that are predetermined during the model’s design phase. In addition, as various sensors operate at distinct frequencies, creating varying time-series lengths, the models’ inference performance is directly dependent on the sensor. As a result, they are not easily adaptable to other devices that work on lower frequencies than the ones used during training. In contrast, recurrent neural networks (RNNs), which are appropriate for time-series data due to their memory ability, can receive different-sized input, unlike other types of neural networks [31,32,33]. However, the interpretability of deep learning models, in general, is limited [34,35,36]. Therefore, when new types of falls arise, they are only recognizable once the model is re-trained with more and newly collected data.

In contrast to the above-mentioned pipelines, heuristic-based methods present low complexity, while they can be easily adapted to the new data by changing or adding functions and thresholds. Due to these capabilities, many machine learning approaches are combined with this type of technique, aiming to reduce the computational cost [37]. In addition, recent studies focused on information retrieval, subsequence search, and similarity measurements of time series, aiming to automatically detect both normal and abnormal patterns, with an extensive application on the task of fall detection [38,39]. To this end, this article proposes an accelerometer- and threshold-based fall detection algorithm using the timestamp of each measure while performing on different frequencies. Furthermore, the presented method detects more than one fall in a time series of sum vector magnitudes, making it ideal for analyzing more extensive data, unlike machine and deep learning models, which classify the input between fall and no-fall classes. Finally, our framework shows its adaptability to work on various acceleration devices as demonstrated during its evaluation in different sets of devices and datasets. Our main contributions are summarized as follows:

- A heuristic-based human fall detection algorithm that uses as input the acceleration information provided at different frequencies to recognize an accident. In addition, more than one event can be detected in a time series of data.
- A method capable of working on various types of sensors.
- An open-source (https://github.com/smoutsis/fall_detection_through_acceleration_data (accessed on 10 September 2023)) and flexible code, provided in two programming languages, Python and C, aiming to facilitate future researchers.

The rest of this article is organized as follows. Section 2 contains the related literature on human fall detectors. Section 3 introduces the methodology, including the two parts of the proposed pipeline, i.e., fall detection and verification. Section 4 contains our experimental protocol and discusses the method’s outcomes, while in Section 5, the discussion part presents a comprehensive analysis and interpretation of the results. Finally, Section 6 concludes this article and gives our plans.

## 2. Related Work

Different approaches have been proposed to address the task of human fall detection. An extensive review, including comparisons among different pipelines, is available in [40]. Yet, our related section consists of two parts, *viz.*, machine learning- and heuristic-based techniques. However, we must note that each of the presented works utilizes data from wearable devices, mostly acceleration sensors.

### 2.1. Machine Learning-Based Fall Detectors

In contrast to techniques where the patterns are defined heuristically, approaches belonging to this category learn the corresponding fall patterns during the training process [27]. Nevertheless, many experiments and data are required to find the appropriate models and relative parameters to tackle the problem unbiasedly. Shawen et al. introduce a framework that automatically detects falls using the sum vector magnitudes of a 5-s window originating from a mobile phone [41]. The predictions of four machine learning models, namely, random forest, gradient boosting, SVM, and XGBoost, are combined for the final decision. Similarly, in [42], three models, *viz.*, a *k*-NN, an SVM, and a neural network, are evaluated regarding their complexity as employed on a wearable device. The models were trained based on a feature vector with a length of 12, extracted through two equal segments of a 3-s window provided by an accelerometer. This data transformation is the main reason every model is lightweight, achieving high accuracy and reaching a score near 98%. Utilizing the sum vector magnitudes of acceleration data in a rule-based algorithm and a CNN, a low-cost and easily applied framework for older people in nursing homes is proposed by the authors in [7]. Between these two, the latter performs better on the three public datasets evaluated. However, the former is chosen, as it produces remarkable accuracy scores, is computationally lighter, and has greater applicability. In a similar manner, and aiming to reduce the computational cost, Putra et al. propose a triggered machine learning pipeline. Their method is based on the sum of acceleration data, while the generated outcome is enabled according to the occurring fall peaks. The extracted features, which are the inputs of the tested models, namely, CART, *k*-NN, logistic regression, and SVM, come from three segments, *viz.*, the pre-peak part, the peak, and the post-peak. A threshold-based approach, similar to [29], is proposed in [37]. Features are extracted according to peaks, while four models are evaluated, *viz.*, a feed-forward neural network, an SVM, a decision tree, and a rule-based system. Moreover, the hidden Markov models (HMMs) handle the task of human activity recognition and the subcategory of fall detection with efficiency and interpretability, due to their natural modeling traits for time-series data [22,43,44]. In [43], the problem is treated as a recognition between the various types of accidents and daily actions, instead of binary classification, and efficiency is tackled by single-state HMM and fixed-number-of-state HMM. Similarly, Hiu et al. present a novel technique based on HMM, focusing on explanation and scalability. Partitioning and state generalization among activities is achieved by drawing inspiration from speech recognition, while the interoperability of the underlying structure of human actions seems to be better in HMM than in neural networks [44].

### 2.2. Heuristic-Based Fall Detectors

Methods belonging to this category define the data pattern needed for detecting a possible human fall. Kangas et al. [45] propose three algorithms that differentiate specific parts of a fall, particularly the beginning, the falling velocity, the fall impact, and the posture after the fall. Their input from an accelerometer, i.e., sum vector magnitudes, and data from three different body parts, the head, the waist, and the wrist, were tested. iFall detects accidents based on upper and lower thresholds, while false positives are reduced using position data and user feedback [46]. The proposed method uses information from a three-axis accelerometer adjusted on user characteristics, such as height, weight, and activity levels. Finally, an alarm is sent through an Android application. Similarly, F2D recognizes an event according to upper and lower thresholds, yet, the information of the fall’s rebound, the residual movement, and the location are also applied [47]. Casilari and Oviedo-Jiménez [48] find that a system’s performance improves if more than one sensor is used. Towards this goal, four algorithms, *viz.*, iFall, basic threshold monitoring, fall index [49], and two-phase detection [50], are tested in their framework. iFall is shown to outperform the other techniques. A threshold-based pipeline that utilizes differential acceleration data instead of the sum of acceleration is proposed in [19]. The sensors are placed to show solely that their direction is known without the user’s contribution [51]. By discretizing a fall event into four parts, *viz.*, fall’s beginning, impact, aftermath, and orientation change, the authors in [51] detect elderly falls through acceleration data in a MATLAB mobile application. An alarm is generated if the algorithm locates these four parts in a consecutive sequence and in a specific time interval.

## 3. Methodology

**Figure 1:**
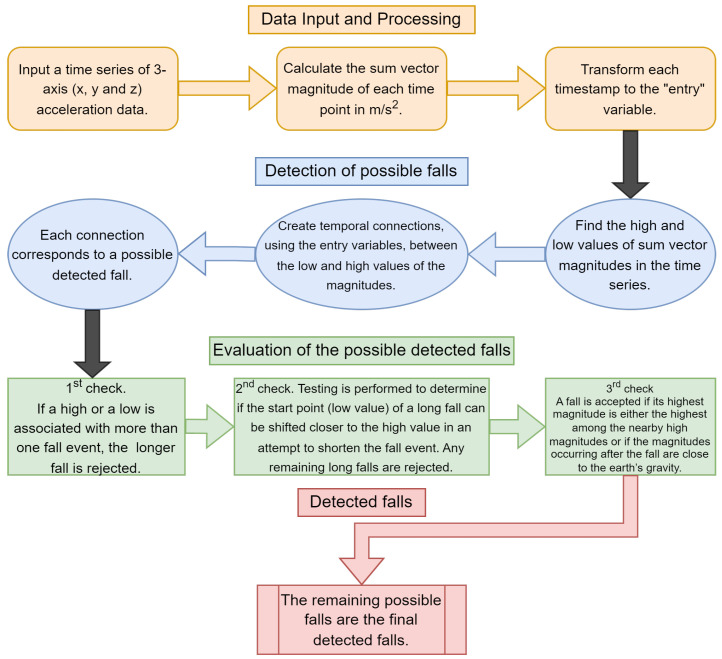
The system’s architecture is distinguished into three stages. The initial step (yellow boxes), including data processing, comes first. Subsequently, potential fall events are identified (blue boxes), while a series of sequential evaluations are performed in each possible fall during the third phase (green boxes). Lastly comes the outcome (red box), which gives the detected fall incidents.

The following section describes the proposed accelerometer- and heuristic-based algorithm for detecting human falls. We start with an overview of the preliminary information needed, and the implementation details for our two-part system, as represented in Figure 1, are given subsequently.

### 3.1. Fall Detection Foundation

In our effort to implement an algorithm that detects falls solely based on data provided by an accelerometer, the limitation of orientation information arises. Consequently, we rely on the sum vector magnitude of x, y, and z measurements (Equation (1)), which remains constant regardless of the sensor’s orientation. As various terms, such as the norma, sum of acceleration, acceleration, and magnitude, are also used to describe the sum vector magnitudes, in this work, we will refer to this measurement as the magnitude:

(1) $${\mathrm{Sum}\mathrm{vector}\mathrm{magnitude}}_{n}={\mathrm{Norma}}_{n}={\mathrm{Acceleration}}_{n}=\sqrt{x_{n}^{2}+y_{n}^{2}+z_{n}^{2}}$$

**Figure 2:**
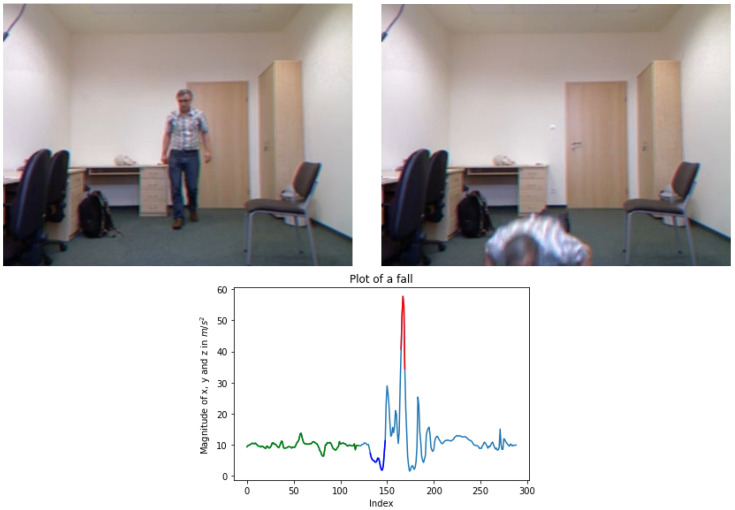
Representative images of a human fall from the UR dataset [10] and the sum vector magnitudes of x, y, and z measured in ${m/s}^{2}$. During an accident (**right image**), a sequence of magnitudes that consists of low values at the beginning (plot’s blue part) and then steep highs (plot’s red part) is generated. As shown, the measurements are close to the earth’s gravity (plot’s green part) as the subject walks (**left image**).

**Figure 3:**
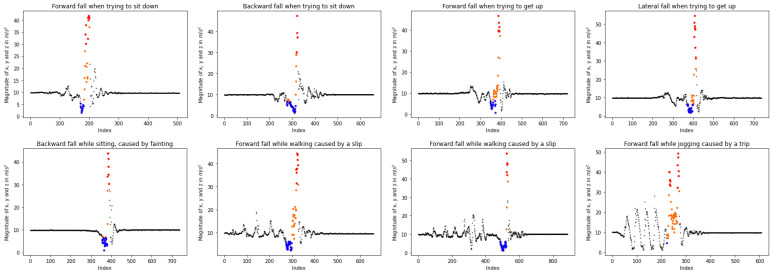
Time-series of sum vector magnitudes in various types of fall, *viz.*, “Forward fall when trying to sit down”, “Backward fall when trying to sit down”, “Forward fall when trying to get up”, “Lateral fall when trying to get up”, “Backward fall while sitting, caused by fainting”, “Forward fall while walking caused by a slip”, “Forward fall while walking caused by a slip” and “Forward fall while jogging caused by a trip” from KFall dataset [21]. The colored dots correspond to falls. More specifically, the blue are the low values of magnitudes, the red are the high values, and the orange ones denote the intermediates.

According to the literature [29,46,47,51] and the visualization of human falls (see Figure 2) during an accident, the sum vector magnitudes generated from the accelerometer data produce a plot similar to the one depicted in Figure 2. The two main parts that imply an event are the low and high values of the magnitudes as shown by the blue and red color (see Figure 2), respectively. Moreover, the same pattern, where low magnitudes are followed by highs, is observed in various types of falls as illustrated in Figure 3. Regarding the measurements after an accident, very small changes are noted. When this initial phase passes, the magnitudes appear to stabilize at the acceleration of gravity ($g=9.80665\;{m/s}^{2}$), suggesting that the person has fallen and is no longer in motion. However, the pattern generated by the sensor before an incident cannot be precisely defined, as it depends on the subject’s previous actions. For instance, when the person sits prior to a fall, the values remain stable at *g* since no movement occurs (Figure 3, lower left). Furthermore, as the activity intensity increases, measurement fluctuations become more pronounced, as evident in the Figure 3 upper plots, where the person either sits down or gets up before the accident.

In relation to those mentioned above, our pipeline comprises two parts. The first regards the detection of human falls based on the low and high values, while the second part evaluates them according to certain checks on time limitations and their impact compared to the neighboring data. As input, we use the magnitude of a three-axis accelerometer.

### 3.2. Detecting Human Falls

**Figure 4:**
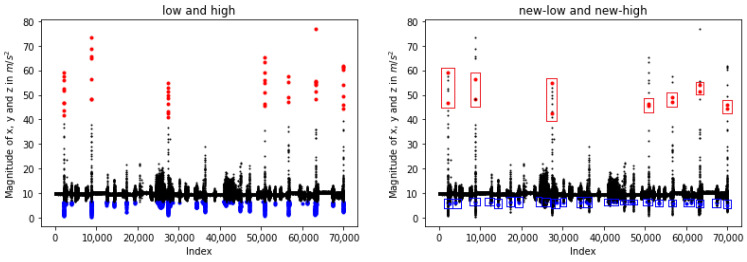
Representation of low and high sum vector magnitudes of x, y, and z accelerations. The blue dots correspond to the lows, while the red ones depict measurements belonging to the highs (**left**). The red rectangles show the set of highs created according to time constraints, while the blue rectangles illustrate the lows (**right**).

At first, each of the low and high magnitudes that reaches a specific level is considered a possible case that should be noted. A representative example is given in Figure 4 (left), wherein seven falls are illustrated. In particular, the red points correspond to the high measurements, the blue ones represent the low, and the black points are the normal conditions. Subsequently, we cluster these values according to their case, i.e., low or high, generating sets of continuous values. There are differentiated based on their time thresholds. Then, each group’s first and last measurements are kept for further processing. Towards this goal, the “entry” variable (Equation (2)) is created and used as a new timestamp. To this end, a set of lows or highs is produced only if the subtraction between two “entries” is bigger than *e* (Figure 4, right).

(2) $${\mathrm{entry}}_{n}=\frac{{\mathrm{timestamp}}_{n}-{\mathrm{timestamp}}_{n-1}}{10}+{\mathrm{entry}}_{n-1}+\frac{100}{\mathrm{Sensor}(\mathrm{Hz})}$$

**Figure 5:**
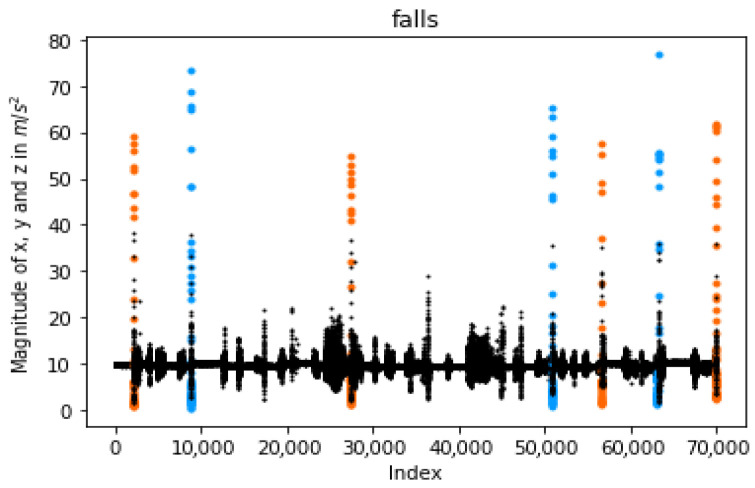
Detected falls as found by our pipeline after connecting highs. The colored dots represent the sum vector magnitudes of x, y, and z corresponding to a human fall.

Using the entry variables, the sets of lows are connected with a group of highs, while these connections correspond to possible falls. Specifically, if a subtraction between a low and a high entry is smaller than fall_duration, then our system considers that these magnitudes correspond to an event. An example is given in Figure 5, wherein the seven falls illustrated by light-blue and orange colors are detected after the proposed connection. The first part of our method is presented in Algorithm 1.
**Algorithm 1** Detecting human falls**Require:** Two lists, magn_list and time_list▹ Lists with the magnitudes of x, y, and z acceleration measurements and the corresponding timestamps**Ensure:** Two lists, fall, and index▹ Lists of lists, where the times and indexes of falls are contained1:low,high,entry← empty lists2:entry.append(0)3:HZ← the frequency of the sensor4:**for** i←1 to length(time_list) **do**5:    entry.append((time_list[i]−time_list[i−1])/10)+entry[i−1]+(100/HZ)6:    **if** magn_list[i]<min_limit **then**7:        low.append(i)8:    **else if** magn_list[i]>max_limit **then**9:        high.append(i)10:    **end if**11:**end for**12:**if** length(high)=0** or **length(low)=0 **then**13:    **return** No Fall14:**end if**15:new_low,new_high← empty lists16:**for** each high, low **do**17:    **if** a high is quite far from the next one **then**▹ Keep the first and last high of a set18:        new_high.append(createasetofhighs)19:    **end if**20:    **if** a low is quite far from the next one **then**▹ Keep the first and last low of a set21:        new_low.append(createasetoflows)22:    **end if**23:**end for**24:fall,index← empty lists25:**for**i←0 to length(new_low) **step** 2 **do**26:    **for** j←0 to length(new_high) **step** 2 **do**27:        **if** a low is close enough to a high **then** ▹ Create a fall with the start point as the low and the finish point as the high28:           fall.append(entry[new_low[i]],entry[new_high[j]])29:           index.append(new_low[i],new_high[j])30:        **end if**31:    **end for**32:**end for**33:**if** length(fall)=0 **then**34:    **return** No Fall35:**else**36:    **return** fall, index37:**end if**

### 3.3. Validating the Detected Accidents

**Figure 6:**
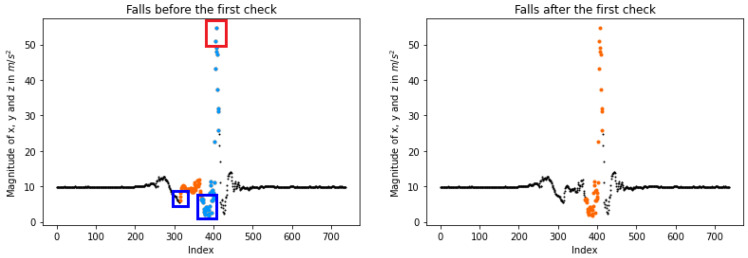
Validating a lateral human fall detection on the KFall dataset [21]. The first check refers to the cases where a low/high is connected with more than one, resulting in multiple falls (**left**). In these cases, the shorter fall is accepted, while the longer is rejected (**right**). Each segmental coloring indicates a different possible fall. In the red boxes the high values are included, while in the blue boxes the low values are incorporated.

Subsequently, the proposed algorithm validates the detected human falls, aiming to accept or reject them. More specifically, we examine if a case participates in multiple events. As shown in Figure 6 (left), the high set is connected with two different low cases and two fall results. In order to address this error, we search for the smaller time range among their connections. This way, only the shorter fall is accepted as depicted in Figure 6 (right).

**Figure 7:**
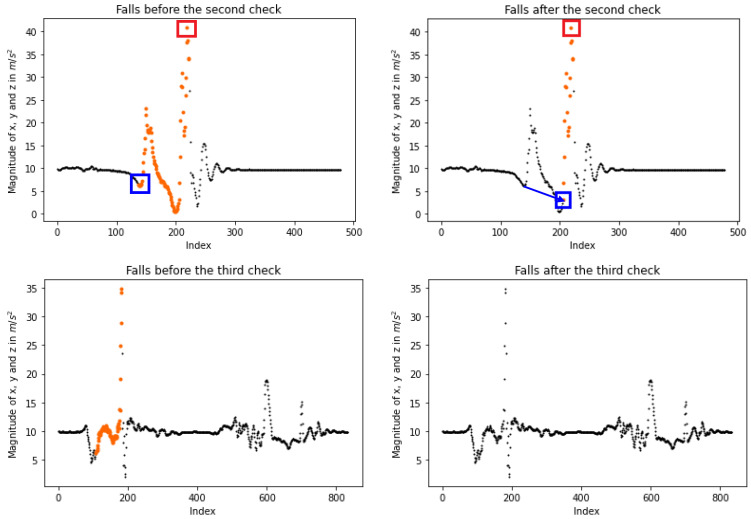
Illustration of the second check after the fall detections. If the starting low point is not close to the high one, it is shifted to another low closer to its peak. This check is depicted in the upper line of the plots, where the initial start point of the left plot (blue box) is transported to another value that is closer to the peak of the fall (red box) as depicted in the right plot. If no low value exists near the high, the detected fall is rejected as it is represented on the plots of the bottom line, where the detected fall of the left plot is not maintained after the third check. The plots in the upper are generated by a “forward fall,” while the plots in the bottom correspond to a “stand, sit and get up” action from the KFall dataset [21]. Each segmental coloring indicates a different possible fall. In the red boxes the high values are included, while in the blue boxes the low values are incorporated.

Afterward, if more continuously low values exist closer to the high ones, each fall’s length is evaluated in order to be shrunk as illustrated in Figure 7 (left). Our technique recognizes a true event when the subtraction between the high-entry, i.e., the end of the fall, and low-entry, i.e., the start of the fall, is greater than fall_limitation. To rectify the long-range accident, the starting point is shifted to the lower value closer to the high one as presented in Figure 7 (right). Additionally, any remaining long fall is rejected due to the fact that a real event occurs in a short period of time [37].

Finally, two more checks are applied to the detected accidents based on the following assumptions:

- Following a fall, the person remains on the ground, and the magnitude of a time series stays close to *g*.
- If more peaks are close to the fall, the highest magnitude should be greater than the neighboring ones.

**Figure 8:**
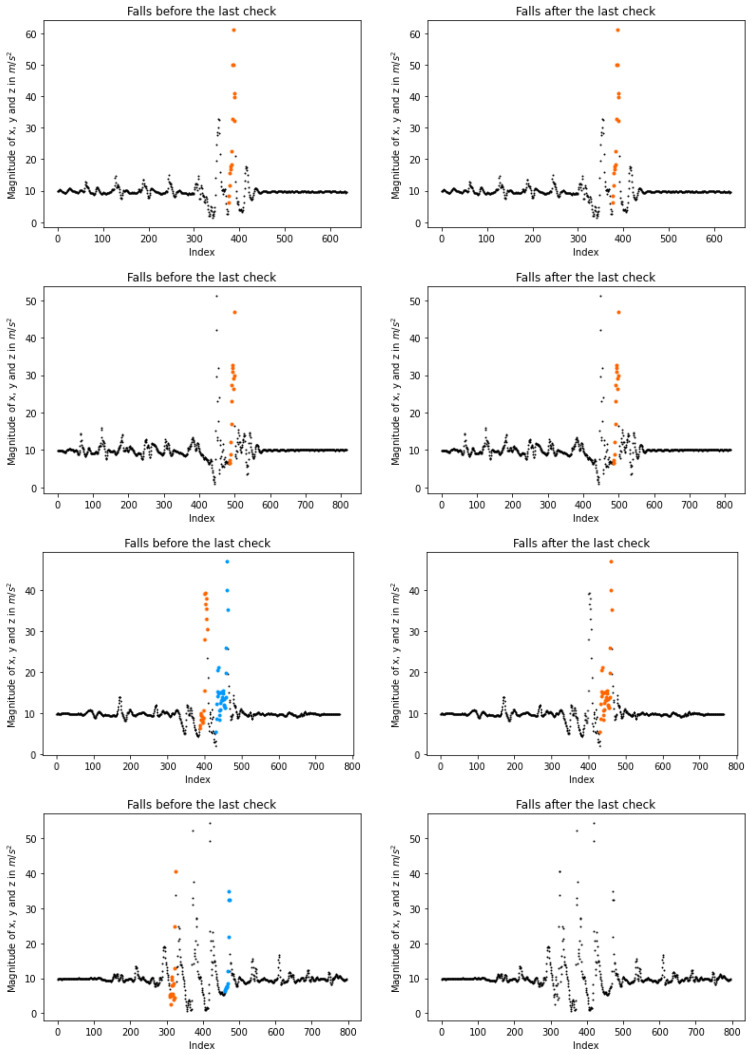
Illustration of the third (last) check after the fall detections. On the **left**, plots show the possible events before the last check, while on the **right**, the corresponding final falls after the check. During our last check, a fall is accepted either if its highest magnitude is also the highest among the close peaks or the magnitudes placed beyond the fall are close to the earth’s gravity *g*. The first line of plots gives an accepted fall when both assumptions are satisfied (the plots correspond to a “forward fall” from KFall [21]), while the second line shows an accepted accident as the fall is close to *g* (the plots correspond to a “backward fall” from KFall). The third line rejects the detected event as neither of the assumptions is valid; however, the second fall is approved, as its peak is higher than any other close to the second event (the plots correspond to a “vertical fall” from KFall). Finally, the last line indicates rejected falls since neither of the assumptions is valid (the plot corresponds to a “stumble while walking” from KFall). Each segmental coloring indicates a different possible fall.

Therefore, an identified event is endorsed if at least one of the aforementioned assumptions is satisfied. Regarding the first, the magnitudes’ average score and the standard deviation are calculated. After the accident, these measurements are taken within a short period of time following the detection through the utilization of entries. The first assumption holds true if the average score is close to *g* while the standard deviation is low, indicating the absence of sharp changes. Concerning the second, it is satisfied if the highest fall magnitude is greater than any other before and after the accident. Figure 8 gives four examples of how the last checks work. Plots on the left depict possible falls before their validation, while the right ones present the final detection. More specifically, the first line illustrates how the system performs when both statements are valid. In the second line of plots, the peak of the possible fall is not the higher one. However, most of the magnitudes next to the fall are close to *g*, so the accident is accepted. Two falls are detected in the third line. Yet, the first one is rejected, as none of the statements are valid, while the second fall is accepted as the highest peak. Finally, two falls are detected in the last line; nevertheless, neither of them is accepted because their peaks are not close to the highest value, and their magnitudes after the event are not close to *g*. Algorithm 2 gives the validation process.
**Algorithm 2** Validating the detected accidents1:**Input:** Four lists, magn_list, time_list, fall and index▹ Lists with the magnitudes of x, y and z accelerations along with the corresponding timestamps and the detected falls from the first part of the algorithm2:**Output:** Two lists, new_fall and new_index▹ Lists of lists, where the times and indexes of the falls are contained3:low,high,entry←emptylists4:g←9.8075:**for** i←0 to length(new_fall) **do**6:    **if** a low or a high participates in more than one fall **then**7:        **keep** the shorter fall and **delete** the longer8:        save the entries in the new_fall list9:        save the indexes in the new_index list10:    **end if**11:**end for**12:**for** each fall in new_fall **do**13:    **if** a fall is long **then**14:        **if** the fall can be shorter **then**15:           swift the start point (low value) of the fall to be closer to the high16:        **else**17:           **delete** the fall18:        **end if**19:    **end if**20:**end for**21:**if length**(new_fall)=0 **then**22:    **return** No Fall23:**end if**24:**for** each fall, magn in new_fall, $magn_{l}ist$ **do**25:    **if** the magnitudes after the fall are close to *g* **or** the peak of the fall is the highest magnitude **then**▹ As the peak is defined, the highest magnitude into the fall range26:        **keep** the fall27:    **else**28:        **delete** the fall29:    **end if**30:**end for**31:**if length**(new_fall)=0 **then**32:    **return** No fall33:**else**34:    **return** new_fall,new_index35:**end if**

## 4. Experiments

The following section evaluates the proposed technique. First, we briefly describe the datasets used for the experimental protocol. Next, the corresponding metrics utilized for measuring the framework’s outcome are given. Finally, an extensive validation of the proposed solution follows.

### 4.1. Datasets

Three publicly available and well-known datasets are selected for our experiments. Among the chosen ones, UR [10] and KFall [21] are selected for validation and threshold determination. Yet, the latter is also used for testing. At last, MMsys (or Cogent) [20] is adopted for testing along with KFall.

#### 4.1.1. Validation Set

UR was created by 9 subjects: it provides red-green-blue, depth, and acceleration data for 3 types of 40 daily actions, *viz.*, lying, sitting, and bending, and 2 types of 30 falls, frontward while standing and frontward while sitting.

**Figure 9:**
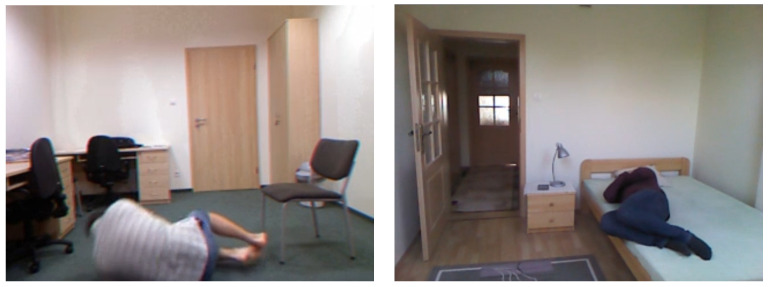
Representative images from the UR dataset [10]. At the **left**, front fall is shown while the subject is sitting. The **right** image depicts the daily action of laying.

Figure 9 shows a fall and daily action snapshots from the dataset’s creation. For our work, only the acceleration data, included in CSV files, are utilized corresponding to a fall or a daily action. However, no information is provided about the time of the measurements. Aiming to tackle this, our pipeline generates timestamps, as it is known that the sensor receives data at a frequency of 60 Hz.

**Figure 10:**
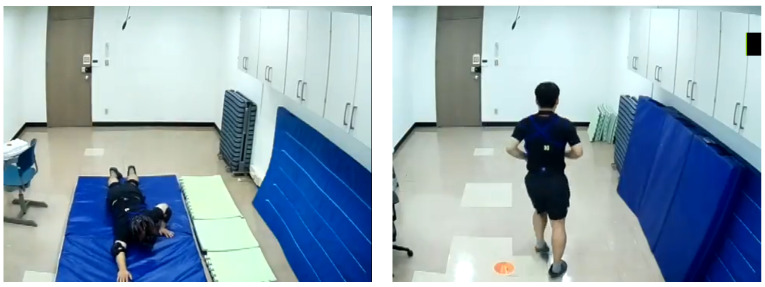
Representative images from the KFall dataset(with permission from [21]). At the **left**, frontward fall caused by a trip while the subject walks. The **right** image depicts the daily action of jogging quickly with a turn—4 m.

KFall was created by 32 subjects who performed 21 types of daily activities: ”walk quickly”, “jog quickly”, “sit down to a chair quickly”, and “lie down on a bed quickly”. Moreover, 15 types of simulated falls are also included, *viz.*, “forward/lateral fall when trying to get up”, “forward/lateral/backward fall while sitting, caused by fainting”, “forward fall while walking/jogging caused by a trip/slip”, and “forward/lateral fall when trying to sit/get down/up”. LPMS-B2, placed on the low back, is used as a sensor for recording since it captures data at a 100 Hz sampling rate and includes a three-axis accelerometer, a three-axis gyroscope, and a three-axis magnetometer. After converting them from *g* ($9.807\;{m/s}^{2}$) to ${m/s}^{2}$, only the acceleration input is employed during our experiments. At last, there are 5075 CSV files, out of which 2729 correspond to daily activity (no fall), and 2347 to a fall. We utilize 20% for validating each sub-class, generating a set of 593 falls and 689 daily actions. Figure 10 shows a fall and a daily action snapshot captured during the dataset generation.

#### 4.1.2. Test Set

MMsys comprises a total of 42 subjects who participated in 7 types of daily actions, *viz.*, “standing”, “lying”, “sitting on the bed”, “sitting on the chair”, “walking”, “crouching”, and “ascending/descending a staircase”. In addition, 6 types of falls: “fall forward”, “fall backward”, “fall right”, “fall left”, “real fall forward”, and “real fall backward” are included.

**Figure 11:**
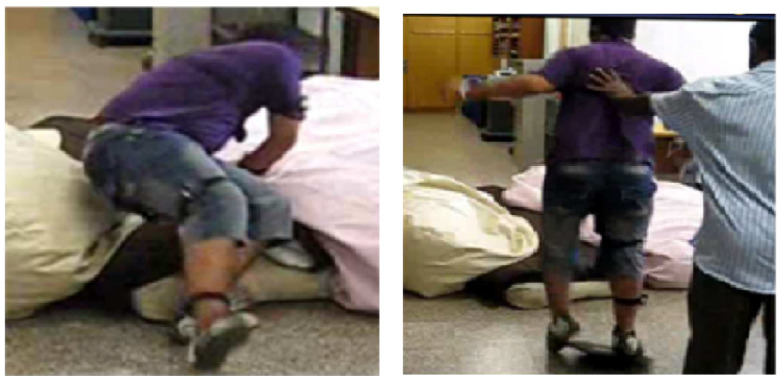
Representative images from the MMsys dataset [20]. A fall is depicted on the **left**, while daily action is shown on the **right**.

Furthermore, one more class was implemented, the “near fall”, which presents cases of balance impairment without resulting in an actual fall. Figure 11 illustrates a fall and a daily action snapshot captured during the dataset’s generation. Concerning the sensors used, two identical SHIMMER devices were adopted. In particular, one was applied to the chest and the other to the thigh of the subject. This way, acceleration and gyroscopic data from both body parts are provided in three axes. Due to the fact that the timestamps or any other information about time are not included, we generated them based on the sensor’s frequency (100 Hz). For each subject, a CSV file, where all the acceleration data are included, is produced, wherein a label indicates the class that the measurement belongs to. In total, MMsys contains 448 falls and 1490 daily activities. At last, the remaining 80% of each sub-class from KFall is also used on testing, creating a test set of 1753 falls and 2040 daily actions.

### 4.2. Thresholds’ Evaluation

UR and KFall are chosen to validate the proposed method, while 5 out of 8 thresholds are evaluated on various values and combinations. The selected values are presented in Table 1. The “min_limit” concerns the maximum value, where a magnitude is considered low, while the “max_limit” indicates the minimum one, where a magnitude is defined as high. “sub_1” defines the time entry distance that should exist between two cases, and it is set to 50 so as to indicate that if the subtraction between two entries exceeds 50 (≃0.5 s), two magnitudes are the end of the previous set and the start of the subsequent one as shown in Figure 4. It is worth noting that a set of highs or lows can consist of one magnitude. Next, “fall_duration” is the maximum value that the subtraction of finish-high and a start-low entries should be so as to consider the connection of these magnitudes as a possible fall. Respectively, “fall_limitation” is the maximum value that should be satisfied, corresponding to the entry difference between the end (last high) and the start of the fall (first low), so as to allow the proposed system to identify a long fall and subsequently validate it. Finally, “dist_1” and “dist_2”, both set to 100, refer to the entry distances before and beyond the fall, wherein highs should be searched. However, during this search, highs are not considered the ones which are greater than “max_limit” but those which are higher than another “max_limit_2”.

### 4.3. Metrics

We used accuracy, sensitivity, and specificity as metrics to validate the proposed fall detector. These are defined by Equations (3)–(5), respectively. Concerning the testing, precision as displayed in Equation (6) is adopted. Both are based on true positive (TP), false positive (FP), true negative (TN), and false negative (FN) detections. TPs are determined as the cases where the system correctly identifies an event, FPs are the incorrectly detected accidents, TNs are the correct non-fall detections, and FNs are the falls that the framework should have recognized but the system did not achieve it. If more events are identified in a time series wherein non-, or a fall, is included, the FPs increase accordingly:

(3) $$\mathrm{Accuracy}=\frac{\mathrm{True}\mathrm{Positives}+\mathrm{True}\mathrm{Negatives}}{\mathrm{True}\mathrm{Positives}+\mathrm{False}\mathrm{Positives}+\mathrm{True}\mathrm{Negatives}+\mathrm{False}\mathrm{Negatives}}$$

(4) $$\mathrm{Sensitivity}=\mathrm{Recall}=\mathrm{True}\mathrm{positive}\mathrm{rate}=\frac{\mathrm{True}\mathrm{Positives}}{\mathrm{True}\mathrm{Positives}+\mathrm{False}\mathrm{Negatives}}$$

(5) $$\mathrm{Specificity}=\frac{\mathrm{True}\mathrm{Negatives}}{\mathrm{True}\mathrm{Negatives}+\mathrm{False}\mathrm{Positives}}$$

(6) $$\mathrm{Precision}=\frac{\mathrm{True}\mathrm{Positives}}{\mathrm{True}\mathrm{Positives}+\mathrm{False}\mathrm{Positive}}$$

Accuracy constitutes the correct detections for non- and fall cases, highlighting their significance. While it is one of the most common metrics used, it should be treated cautiously, as it depends on the applied dataset. For instance, if a pipeline detects only falls when 95 falls and 5 non-falls exist, the accuracy would be 95%. The metric of sensitivity, or also recall, reveals how the system performs concerning positive detections. On the other hand, specificity reflects the algorithm’s ability to handle negative recognitions by determining the rate of correct non-falls detections. The above-mentioned metrics should be considered simultaneously, as each alone can lead to wrong conclusions. Finally, precision is defined as the system’s ability to avoid FPs. This metric is complementary to sensitivity. A high score for both sensitivity and specificity is desirable, as their balance is essential. However, a higher score on the former is preferred over the latter, as it is advantageous to detect more false falls than losing an actual one.

### 4.4. Validation

Various experiments were conducted to determine the thresholds and assess the algorithm’s performance by applying different combinations of values. Table 1 presents five out of the eight thresholds, *viz.*, min_limit, max_limit, fall_duration, fall_limitation and max_limit_2, that are tested. The three remaining thresholds, namely sub_1, dist_1, and dist_2, are set on 50, 100, and 100, respectively. Our validation is divided into two parts: the first comprises fall_limitation and max_limit_2 when initially set to 100 and “max_limit”, respectively. The remaining three thresholds were tested (see Table 2). The second part evaluates the previous contrast values (see Table 3). The first three received the highest values achieved during the first part. Considering that the lowest magnitudes on KFall and UR are 0.11 and 0.05 and the greatest are 67.89 and 111.81, we set the initial validation values of “min_limit” to 2 and “max_limit” to 100. The fall_duration is selected at 100, which corresponds to about one second.

Table 2 gives the results on the validation sets. The first line of scores corresponds to the initialized values of thresholds. In cases where min_limit is less than or equal to 5 and max_limit is larger than 40, very high scores on specificity and very low scores on sensitivity are noticed. This imbalance is observed because highs are characterized only by the elevated points, while lows are classified solely by the very low points. As a result, the connections between them that correspond to falls are restricted, and our system fails in detecting falls, yet performs well in detecting non-falls. Therefore, this fact is reasonable in KFall when the max_limit is over 60, as the highest magnitude in the validation set of KFall is 67.89. However, higher values for max_limit were tested for evaluating the performance on UR. The highest magnitude is 111.81. The imbalance between sensitivity and specificity is reduced as min_limit gets higher and max_limit lower. More specifically, on KFall, the most imbalanced performance, i.e., 85.16% and 87.95% in sensitivity and specificity, respectively, is reached when min_limit is set to 5, max_limit to 35, and fall_duration to 100. Regarding UR, we achieved 80.00% in sensitivity and 85.00% in specificity when min_limit is 6, max_limit is 25, and fall_duration is 100. Due to the fact that the aforementioned performance results are attained at different thresholds, max_limit receives a value that depends on the average score of all the magnitudes in subsequent experiments, aiming to adapt the algorithm each time to the data it receives.

**Figure 12:**
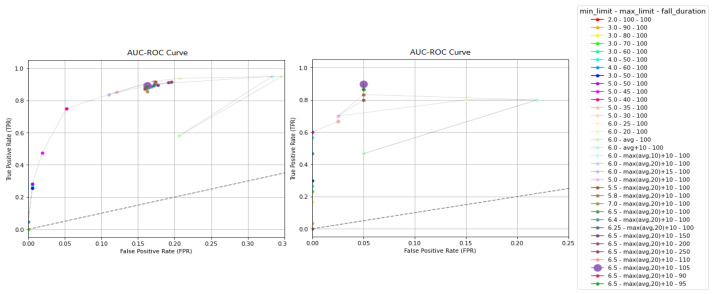
AUC–ROC curves are shown for 32 different combinations of thresholds, including min_limit, max_limit, and fall_duration, while the thresholds fall_limitation and max_limit_2 are set to 100. The left curve corresponds to the results from KFall [21], while the right plot depicts the metrics for UR [10]. The combination of the chosen threshold values is represented by the larger dot.

Furthermore, in Figure 12 the outcomes detailed in Table 2 are given through AUC–ROC curves. In these representations, each combination of thresholds is depicted on a plot, where the y-axis represents the true positive rate (TPR) or sensitivity, while the x-axis corresponds to the false positive rate (FPR), which is calculated as 1−Specificity. In an AUC–ROC curve, a model positioned closer to 1 for TPR and closer to 0 for FPR is considered a better classifier. Regarding KFall (see Figure 12, left), it is observed that the algorithm’s performance is enhanced when the max_limit average score is set to max(avg,20)+10. Additionally, a balance between high TPR and low FPR is established when min_limit is set to 6.5, and fall_duration remains close to 100. On the contrary, very low values for min_limit and high values for max_limit lead to reduced FPR and TPR (Figure 12).

It is worth noting that the highest TPR is attained when the values 6.5, max(avg,20) + 10, and 105 are set for the thresholds min_limit, max_limit, and fall_duration, respectively, as shown in Figure 12. Meanwhile, the FPR remains at 0.05. Consequently, the aforementioned values are chosen in the initial validation phase due to their superior and balanced performance in terms of sensitivity and specificity. This selection is substantiated by the data presented in Table 2 and the AUC–ROC curves of Figure 12, which demonstrate their effectiveness on both KFall and UR.

Next, the two remaining thresholds, fall_limitation and max_limit_2, are examined by keeping constant the first three thresholds at the values and variables that were defined previously. As depicted in Table 3, our metrics on UR are maintained at 90.00% and 95.00% until the fall_limitation is lower than 75. This occurrence is attributed to the fact that max_limit_2 is applied on the data where continuous peaks arise, e.g., when the subject runs or jumps. However, in contrast with KFall, in UR, such actions are not included. Furthermore, max_limit_2 is initially set to be equal to max_limit and was tested only with higher values. Lower ones would not affect our algorithm’s detections since this threshold specifically pertains to neighboring peaks, where the fall should be greater in order to be accepted. Nevertheless, as depicted in the first five lines of Table 3, where higher values were tested, the performance exhibited a decline. Regarding fall_limitation in both datasets, the sensitivity decreases as it receives very low values (70, 65, and 60), causing our system to become more stringent in accepting a fall.

**Figure 13:**
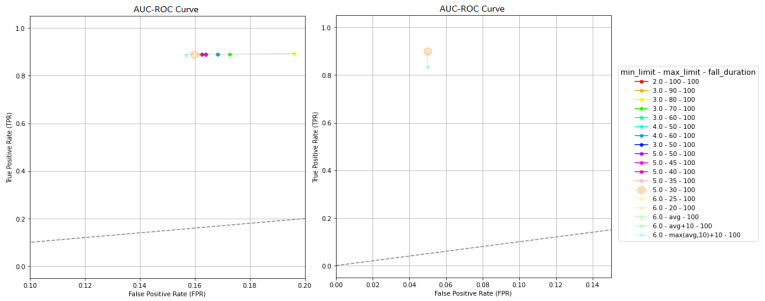
AUC–ROC curves are shown for 18 different combinations of thresholds, including fall_limitation and max_limit_2, while the thresholds min_limit, max_limit, and fall_duration are set to 6.5, max(avg, 20) + 10 and 110, respectively. The left curve corresponds to the results from KFall [21], while the right plot depicts the metrics for UR [10]. The larger dots on the plots represent the combination of selected threshold values.

Respectively, from the AUC–ROC curves, which are depicted in the plots of Figure 13, it is evident that changes in the thresholds of the second validation phase affect the algorithm’s performance less compared to those of the first part, as the various versions are placed close to each other. Particularly, in the curve of UR (Figure 13, right), all the combinations are placed at exactly the same point except for the three cases, where the $fall{\$}_{l}imitation$ is less than 70, while TPR is reduced. Additionally, from the KFall’s curve (Figure 13, left), it is observed that as the max_limit_2 is increased from the max_limit, FPR is also increased without TPR being enhanced. As for the fall_limitation threshold, the algorithm’s performance remains stable for values either lower or higher than 100. However, when it falls below 70, there is a noticeable but minor impact on both TPR and FPR.

Finally, when fall_limitation is set at 85, 80, and 75, the sensitivity on KFall remains unchanged at 89.04%. Yet, the specificity improves from 83.74% to 84.03%, prompting us to choose 85 for fall_limitation, aiming to accept more falls, and set max_limit_2 to be equal to max_limit (=max(avg,20)+10).

### 4.5. Method’s Outcome and Comparative Results

After our validation process, the final values of each threshold as chosen are as follows:

- min_limit=6.5, evaluated.
- max_limit=max(average(magnitudes),20)+10, evaluated.
- sub_1=50, not evaluated.
- fall_duration=105, evaluated.
- fall_limitation=85, evaluated.
- dist_1=100, not evaluated.
- dist_2=100, not evaluated.
- max_limit_2=max_limit, evaluated.

Table 4, wherein the final results on the test sets are depicted, shows a sensitivity of 90.40% and 91.56% on MMsys and KFall, respectively, exhibiting the improved performance of our human fall detector. Additionally, the system proves it is not weak to non-fall actions, as it achieves a specificity score of 93.96% on MMsys and 85.90% on KFall. At the same time, the balance between sensitivity and specificity declares that our framework distinguishes a fall from daily activity. Moreover, the proposed pipeline outperforms the heuristic-based approach of [45] on MMsys, concerning the sensitivity and the rule-based method of [7] on KFall. Regarding the precision, both other pipelines achieve higher rates, implying that FPs are less; yet, it is preferable for a system to detect false events but, at the same time, identify a correspondingly higher number of TPs, especially if the balance between these metrics is maintained. The comparison between the proposed algorithm, the logistic regression described in [29], and the CNN-based in [7] indicates that our framework does not outperform the machine learning approaches. Finally, regarding the part of the place where the sensor is located on the human body, the results on MMsys demonstrate that our system fails to reach high performance results when the sensor is positioned on the thigh instead of the chest. The scores of 76.88%, 62.28%, 81.28%, and 50.00%, for accuracy, sensitivity, specificity, and precision, respectively, declare this fact.

Table 5 and Table 6 present TPs and FPs for each sub-class separately when tested on MMsys and KFall. The aforementioned results permit us to understand where the algorithm performs well and where it does not. In Table 7, the results from Table 5 and Table 6 are compressed by displaying the set of sub-classes within the corresponding range of false rates. In a total of 50 sub-classes, in both test sets, the proposed pipeline does not make mistakes in 15 sub-classes, while the false rate is very small, from 0.1% to 5%, in the other 16. Moreover, it performs well in six more sub-classes, showing a false rate between 5.1% and 10%, while in the other eight, it ranges from 10.1 to 15%. The following five sub-classes constitute the weak aspect of our system. In particular, “Near fall” on MMsys reaches a false rate of 46.99%. “Stumble while walking” on KFall reaches a score of 51.26%. “Forward falls while jogging caused by a trip” on KFall obtains a 60.71% false rate. Similarly, “Sit a moment trying to get up and collapse into a chair” on KFall obtains a score of 69.23%, and finally, “Gently jump when trying to reach an object” on KFall achieves a 82.50% false rate.

Lastly, in “False-2” at Table 5 and Table 6, we present every wrong detection that occurred before our last check. When it is not applied, the performance is reduced, particularly in the non-fall sub-classes, where the data patterns are similar to those of falls. It is worth noting that in KFall, when the last check is missing, the action “Gently jump trying to reach an object” presents false detections ranging from 99 to 109. Similarly, for “Jog normally with turn (4 m)”, these increase from 3 to 60; for “Jog quickly with turn (4 m)”, from 5 to 76; and for “Stumble while walking”, they rise from 59 to 84. Similarly, in MMsys, when the last check is missing, the “Near fall” sub-class, shows a score ranging from 85 to 92. Additionally, for “Ascending and Descending a staircase”, the wrongs increase from 4 to 19.

## 5. Discussion

This work proposes an easily adjusted fall detector capable of distinguishing various types of accidents from different daily actions. Moreover, our system is open sourced in two programming languages, Python and C, so that it can adapt to multiple systems. Additionally, there are no limitations in the acceleration device used since the algorithm has already been applied in a dataset created by a 60 Hz sensor, i.e., UR, and two more i.e., KFall and MMsys, where 100 Hz differential sensors were utilized. Our framework is based only on the acceleration data and the corresponding timestamps. Therefore, any device with a three-axis acceleration input can be used without the need for other similar sensors, e.g., gyroscope, or magnetometer. Finally, its flexibility is shown in the data length; it can detect more than one fall in a time series of magnitudes, as depicted in Figure 5, where the input is the whole time series and the proposed system detects seven falls.

**Figure 14:**
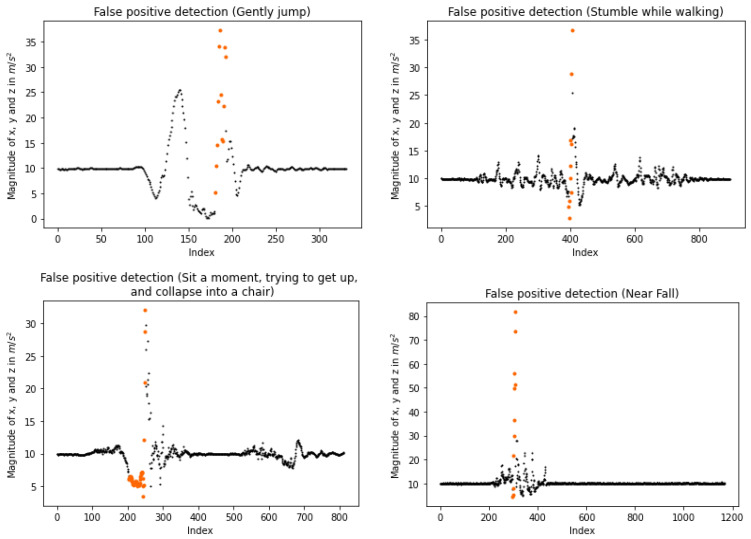
False positive detections. At the upper left, a false positive detection on the “Gently jump” sub-class of KFall dataset (with permission from [21]). At the top right, a false positive on the “Stumble while walking” sub-class of KFall. In the lower left, a false positive on the “Sit a moment, trying to get up, and collapse into a chair” sub-class of KFall. At the bottom right, a false positive on the “Near fall” sub-class of MMsys (with permission from [20]). Each segmental coloring indicates a different possible fall.

Regarding its performance, Table 5, Table 6 and Table 7 declare that it can distinguish various types of falls ranging from daily actions. As shown by the results, it performs highly in 45 sub-classes out of 50. Nevertheless, we acknowledge that our algorithm presents a drawback that lies in these five remaining sub-classes. In particular, the daily actions “Gently jump”, “Stumble while walking”, “Sit a moment, trying to get up, and collapse into a chair”, and “Near fall” are mostly misclassified as falls, while the cases of “Forward fall while jogging caused by a trip” is mostly detected as non-falls events. These four sub-classes are incorrectly classified as positive cases because their magnitude patterns resemble those corresponding to actual accidents. As shown in Figure 14, which illustrates false positive detections, low magnitude values are positioned closely to high values. Consequently, connections between lows and highs are formed, and these connections are not rejected in the second part of the evaluation, resulting in false cases of falls. However, from the aforementioned four sub-classes, that which requires attention is “Gently jump”, as the other three actions are similar to a fall, so a possible alarm would be beneficial, especially for elderly people. Regarding the “Gently jump” activity before the peak that corresponds to an accident, another lower peak is depicted (see upper left plot of Figure 14) that could be used by an extra check so as to reject these possible falls.

**Figure 15:**
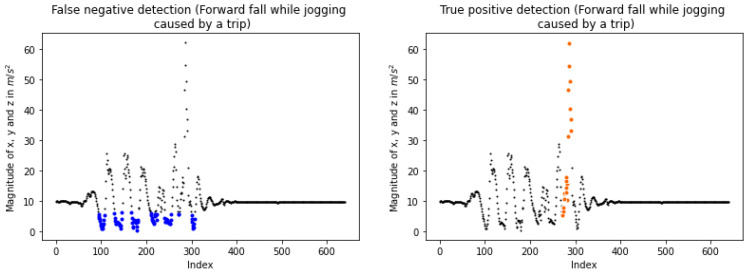
False negative and true positive detections. At the left, a false negative detection on the “Forward fall while jogging caused by a trip” sub-class of KFall (with permission from [21]). The blue dots correspond to the continuously low values of magnitudes which are responsible for not establishing possible falls. At the right, the fall (orange colored segment) is detected by the algorithm as the sub_1 threshold changes from 50 to 10.

Additionally, in the “Forward fall while jogging caused by a trip” sub-class, falls are not detected because of the continuously low magnitudes as depicted in the left plot of Figure 15. This is owed to jogging, which produces low sets that do not lead to a connection between a low and a high for positive recognition. One solution would be to make sub_1 smaller, from 50 to 10, aiming for generating smaller sets of high and low cases; This way, the fall depicted in Figure 15 is detected by the algorithm as illustrated in the right plot. Moreover, the sensitivity is increased to 96.06% at the test set of KFall and detects all the falls in “Forward fall while jogging caused by a trip”. However, the specificity is totally decreased, to 70.94%. To overcome this conflict, a better approach would be to consider the total of lows and highs included in a set and create one more check. Moreover, our method performs well when considering the “False” and “False-2” columns of Table 5 and Table 6. In particular, the last check corrects many FPs without greatly affecting the algorithm’s outcome when detecting actual events.

Finally, as far as the possible disturbance on the signals is concerned, it is worth noting that the proposed algorithm utilizes both the low and high values of magnitudes. Consequently, instantaneous incorrect measurements are unlikely to significantly affect the algorithm’s performance. However, it is important to acknowledge that further research is required in this area, as the used datasets do not include data with disturbance on the signals.

## 6. Conclusions and Future Work

This article proposes a heuristic-based algorithm for detecting falls, using as input the magnitudes of the x, y, and z axes provided by an accelerometer. When the incoming data arrive, our pipeline initially detects a set of possible events and subsequently evaluates them via three checks, aiming to determine the final decisions. The system is designed to be independent of both the sensor’s operating frequency and the size of the received input, while it also can detect multiple falls in long time series without the necessity of separating the input into smaller windows. Its effectiveness is evaluated, as high sensitivities are reached on MMsys and KFall public datasets, reaching 90.40% and 91.56% scores, respectively. In addition, its specificity scores of 93.96% and 85.90% demonstrate further its ability to distinguish falls from daily actions. Our future plans include the development of a generalized buffer, capable of dynamically adjusting the algorithm’s behavior through the thresholds’ modification. This way, we intend to allow the system to be balanced between the two classes or be more sensitive to either falls or non-falls, thus enabling users to adapt its usage according to specific system requirements and providing a tailored approach suiting distinct application needs.

## Figures and Tables

**Table 1:** The main thresholds which are utilized on the proposed algorithm are 8, *viz.*, min_limit, max_limit, sub_1, fall_duration, fall_limitation, dist_1, dist_2 and max_limit_2. Five of them are evaluated, aiming to find the combination of thresholds needed for achieving higher performance in the validation set. The remaining three are set during the implementation of the algorithm.

Index	Description	Threshold	Evaluated
1	The upper limit threshold for classifying magnitudes is low.	min_limit	YES
2	The low limit threshold for classifying magnitudes as high	max_limit	YES
3	The minimum subtraction, of the entries, that two lows/highs must have to be considered a different set of lows/highs	sub_1 =50	NO
4	The maximum subtraction, of the entries, that a set of highs with a set of lows should have to be considered a fall	fall_duration	YES
5	The minimum subtraction of the entries that start with the end of a fall should have to be defined as long	fall_limitation	YES
6, 7	The entry distance before and after the fall, where the algorithm should search for highs	dist_1 =100, dist_2 =100	NO
8	The low limit threshold for classifying neighboring, to the fall, magnitudes as high	max_limit_2	YES

**Table 2:** Results on the first part of validation. Accuracy, sensitivity, and specificity on KFall [21] (validation set) and UR [10] datasets are presented, while the thresholds min_limit, max_limit, and fall_duration are evaluated. The thresholds of fall_limitation and max_limit_2 are set on 100 and max_limit, respectively. The bolds indicate the chosen variable and values for the thresholds.

Thresholds			KFall Validation Set			UR		
Min_Limit	Max_Limit	Fall_Duration	**Accuracy**	**Sensitivity**	**Specificity**	**Accuracy**	**Sensitivity**	**Specificity**
2	100	100	53.74%	0.00%	100.00%	57.14%	0.00%	100.00%
3	90	100	53.74%	0.00%	100.00%	58.57%	3.33%	100.00%
3	80	100	53.74%	0.00%	100.00%	64.29%	16.67%	100.00%
3	70	100	53.74%	0.00%	100.00%	67.14%	23.33%	100.00%
3	60	100	55.93%	4.72%	100.00%	68.57%	26.67%	100.00%
4	50	100	65.91%	26.98%	99.42%	81.43%	56.67%	100.00%
4	60	100	55.93%	4.72%	100.00%	77.14%	46.67%	100.00%
3	50	100	65.29%	25.63%	99.42%	70.00%	30.00%	100.00%
5	50	100	66.46%	28.16%	99.42%	82.86%	60.00%	100.00%
5	45	100	74.65%	47.39%	98.11%	82.86%	60.00%	100.00%
5	40	100	85.65%	75.04%	94.78%	84.29%	66.67%	97.50%
5	35	100	86.66%	85.16%	87.95%	84.29%	66.67%	97.50%
5	30	100	87.05%	91.91%	82.87%	85.71%	70.00%	97.50%
6	25	100	85.97%	93.76%	79.28%	82.86%	80.00%	85.00%
6	20	100	79.00%	94.94%	65.37%	78.87%	80.00%	78.05%
6	avg	100	69.58%	58.01%	79.40%	74.29%	46.67%	95.00%
6	avg+10	100	79.70%	94.94%	66.67%	78.87%	80.00%	78.05%
6	max(avg,10)+10	100	79.70%	94.94%	66.67%	78.87%	80.00%	78.05%
6	max(avg,20)+10	100	86.19%	90.22%	82.73%	90.00%	83.33%	95.00%
6	max(avg,20)+15	100	86.51%	83.64%	88.97%	90.00%	83.33%	95.00%
5	max(avg,20)+10	100	87.05%	91.91%	82.87%	85.71%	70.00%	97.50%
5.5	max(avg,20)+10	100	86.66%	91.40%	82.58%	88.57%	80.00%	95.00%
5.8	max(avg,20)+10	100	86.35%	90.56%	82.73%	90.00%	83.33%	95.00%
7	max(avg,20)+10	100	84.63%	85.67%	83.74%	92.86%	90.00%	95.00%
6.5	max(avg,20)+10	100	85.96%	88.53%	83.74%	92.86%	90.00%	95.00%
6.4	max(avg,20)+10	100	85.73%	88.70%	83.16%	92.86%	90.00%	95.00%
6.25	max(avg,20)+10	100	85.80%	89.21%	82.87%	91.43%	86.67%	95.00%
6.5	max(avg,20)+10	150	85.57%	89.38%	82.29%	92.86%	90.00%	95.00%
6.5	max(avg,20)+10	200	85.66%	91.23%	80.87%	92.86%	90.00%	95.00%
6.5	max(avg,20)+10	250	85.57%	91.57%	80.41%	92.86%	90.00%	95.00%
6.5	max(avg,20)+10	110	86.12%	89.04%	83.60%	92.86%	90.00%	95.00%
**6.5**	**max(avg, 20) + 10**	**105**	**86.19%**	**89.04%**	**83.74%**	**92.86%**	**90.00%**	**95.00%**
6.5	max(avg, 20) + 10	90	85.49%	87.18%	84.03%	91.43%	86.67%	95.00%
6.5	max(avg, 20) + 10	95	85.96%	88.36%	83.89%	91.43%	86.67%	95.00%

**Table 3:** Results of the second part of validation. The metrics accuracy, sensitivity, and specificity on KFall [21] (validation set) and UR [10] datasets are represented, while the thresholds fall_limitation and max_limit_2 are evaluated. The thresholds min_limit, max_limit, and fall_duration are set on 6.5, max(average(magnitudes),20)+10 and 105, respectively, as these values (and variable) arise from the first part. The bolds represent the chosen variable and value for the thresholds.

Thresholds		KFall Validation-Set			UR		
Fall_Limitation	Max_Limit_2	**Accuracy**	**Sensitivity**	**Specificity**	**Accuracy**	**Sensitivity**	**Specificity**
100	max_limit	86.19%	89.04%	83.74%	92.86%	90.00%	95.00%
100	max_limit+5	85.88%	89.04%	83.16%	92.86%	90.00%	95.00%
100	max_limit+10	84.45%	89.21%	80.38%	92.86%	90.00%	95.00%
100	max_limit+6	85.65%	89.04%	82.73%	92.86%	90.00%	95.00%
100	max_limit+1	86.19%	89.04%	83.74%	92.86%	90.00%	95.00%
105	max_limit	86.12%	89.04%	83.60%	92.86%	90.00%	95.00%
110	max_limit	85.88%	89.04%	83.16%	92.86%	90.00%	95.00%
115	max_limit	86.12%	89.04%	83.60%	92.86%	90.00%	95.00%
120	max_limit	86.12%	89.04%	83.60%	92.86%	90.00%	95.00%
125	max_limit	86.12%	89.04%	83.60%	92.86%	90.00%	95.00%
95	max_limit	86.19%	89.04%	83.74%	92.86%	90.00%	95.00%
90	max_limit	86.27%	89.04%	83.89%	92.86%	90.00%	95.00%
**85**	max_limit	**86.35%**	**89.04%**	**84.03%**	**92.86%**	**90.00%**	**95.00%**
80	max_limit	86.35%	89.04%	84.03%	92.86%	90.00%	95.00%
75	max_limit	86.35%	89.04%	84.03%	92.86%	90.00%	95.00%
70	max_limit	86.35%	88.70%	84.33%	90.00%	83.33%	95.00%
65	max_limit	86.35%	88.70%	84.33%	90.00%	83.33%	95.00%
60	max_limit	86.27%	88.53%	84.33%	90.00%	83.33%	95.00%

**Table 4:** Accuracy, sensitivity, specificity, and precision of our method on MMsys and KFall test datasets, after determining the thresholds. For comparison reasons, the results from [7,29] on MMsys and KFall, respectively, are also provided.

		Accuracy	Sensitivity	Specificity	Precision
MMsys [20]	Proposed Algorithm (from chest)	93.14%	90.40%	93.96%	81.82%
	Proposed Algorithm (from thigh)	76.88%	62.28%	81.28%	50.00%
	IMPACT + POSTURE [29,45]	-	87.60%	-	90.90%
	EvenT-ML [29]	-	98.10%	-	97.20%
KFall [21]	Proposed Algorithm	88.51%	91.56%	85.90%	84.79%
	Rule Based [7]	84.89%	80.66%	-	85.35%
	CNN [7]	93.44%	93.30%	-	93.43%

**Table 5:** Results of the proposed algorithm in each sub-class of MMsys [20], separately. The “True” column shows how many of the detections are correct for each class on MMsys, while the “False” represents the wrong ones. An actual accident concerns the true positive and true negative in a fall and in a non-fall class, respectively. However, while a false corresponds to a false positive in a non-fall class and a false negative in a fall class, some of the false in positive classes correspond to a false positive, as the algorithm detects more than one fall in a time series where only one fall is depicted. In the column “False-2”, we have the false detections if the last evaluation of the possible falls is not applied by the algorithm.

Label	Class	Totals	True	False	False-2
1	Standing	129	129	0	0
2	Fall forward	128	119	9	10
3	Lying	322	321	1	1
4	Sitting on a bed	256	256	0	0
5	Sitting on a chair	192	192	0	0
6	Fall backward	64	56	8	8
7	Near fall	183	98	85	92
8	Walking	127	127	0	0
9	Crouching	127	127	0	0
10	Fall right	64	57	7	7
11	Fall left	64	56	8	8
12	Real fall forward	64	59	5	5
13	Real fall backward	64	58	6	3
15	Ascending and Descending a staircase	154	150	4	19

**Table 6:** Results of the proposed algorithm in each sub-class of the KFall [21] dataset, separately. The “True” column shows how many of the detections are correct for each class, while the “False” column represents the wrong ones. Note that an actual event is true positive and true negative in a fall and in a non-fall class, respectively. However, while a false corresponds to a false positive for the non-fall class and a false negative for the fall class, some of the false positive classes correspond to false positive, as the algorithm has detected more than one fall in a time series where only one fall is depicted. In the column “False-2”, we have the false detections if the last evaluation of the possible falls is not applied by the algorithm.

Label	Class	Total	True	False	False-2
1	Stand for 30 s	24	24	0	0
2	Stand, slowly bend the back with or without bending at knees, tie shoe lace, and get up	117	117	0	0
3	Pick up an object from the floor	117	117	0	0
4	Gently jump (try to reach an object)	120	21	99	109
5	Stand, sit to the ground, wait a moment, and get up with normal speed	117	104	13	13
6	Walk normally with turn (4 m)	113	113	0	0
7	Walk quickly with turn (4 m)	115	113	2	4
8	Jog normally with turn (4 m)	117	114	3	60
9	Jog quickly with turn (4 m)	113	108	5	76
10	Stumble while walking	119	60	59	84
11	Sit on a chair for 30 s	24	24	0	0
12	Sit on the sofa (back is inclined to the support) for 30 s	24	24	0	0
13	Sit down to a chair normally, and get up from a chair normally	111	111	0	0
14	Sit down to a chair quickly, and get up from a chair quickly	116	107	9	9
15	Sit a moment, trying to get up, and collapse into a chair	117	36	81	81
16	Stand, sit on the sofa (back is inclined to the support), and get up normally	114	114	0	0
17	Lie on the bed for 30 s	24	23	1	1
18	Sit a moment, lie down on the bed normally, and get up normally	114	114	0	0
19	Sit a moment, lie down on the bed quickly, and get up quickly	111	111	0	0
20	Forward fall when trying to sit down	119	112	7	5
21	Backward fall when trying to sit down	120	117	3	3
22	lateral fall when trying to sit down	120	116	4	4
23	Forward fall when trying to get up	116	112	4	0
24	lateral fall when trying to get up	117	106	11	12
25	Forward fall while sitting, caused by fainting	118	106	12	6
26	lateral fall while sitting, caused by fainting	116	99	17	17
27	Backward fall while sitting, caused by fainting	118	115	3	3
28	Vertical(forward) fall while walking caused by fainting	115	114	1	3
29	Fall while walking, use of hands to dampen fall, caused by fainting	114	110	4	6
30	Forward fall while walking caused by a trip	114	112	2	2
31	Forward fall while jogging caused by a trip	112	43	69	59
32	Forward fall while walking caused by a slip	120	117	3	2
33	Lateral fall while walking caused by a slip	118	114	4	7
34	Backward fall while walking caused by a slip	118	112	6	4
35	Walk upstairs and downstairs normally (5 steps)	102	101	1	1
36	Walk upstairs and downstairs quickly (5 steps)	111	98	13	17

**Table 7:** Distribution of false rates in MMsys [20] and KFall [21]. Each rate range depicts how many sub-classes have the proposed algorithm, while a false rate is how many are within the corresponding range. On 15 sub-classes, there are no wrong detections. On 16 sub-classes, the false rate is very small, 0.1–5%, and for the other 8, it is smaller than 15%. The provided method fails only in 5 sub-classes, as the false rate is greater than 45.1%.

	False Rate Ranges								
	**0**	**0.1–5**	**5.1–10**	**10.1–15**	**45.1–50**	**50.1–55**	**60.1–65**	**65.1–70**	**80.1–85**
MMsys	5	2	3	3	1	0	0	0	0
KFall	10	14	3	5	0	1	1	1	1
Total	15	16	6	8	1	1	1	1	1

## Data Availability

The original contributions presented in the study are included in the article; further inquiries can be directed to the corresponding author/s.
